# Crosstalk Between Autophagy and the cGAS–STING Signaling Pathway in Type I Interferon Production

**DOI:** 10.3389/fcell.2021.748485

**Published:** 2021-11-29

**Authors:** Kunli Zhang, Sutian Wang, Hongchao Gou, Jianfeng Zhang, Chunling Li

**Affiliations:** ^1^ Institute of Animal Health, Guangdong Academy of Agricultural Sciences, Key Laboratory of Livestock Disease Prevention of Guangdong Province, Scientific Observation and Experiment Station of Veterinary Drugs and Diagnostic Techniques of Guangdong Province, Ministry of Agriculture and Rural Affairs, Guangzhou, China; ^2^ State Key Laboratory of Livestock and Poultry Breeding, Guangdong Key Laboratory of Animal Breeding and Nutrition, Institute of Animal Science, Guangdong Academy of Agricultural Sciences, Guangzhou, China; ^3^ Maoming Branch, Guangdong Laboratory for Lingnan Modern Agriculture, Guangdong, China

**Keywords:** autophagy, CGAS, STING, type I IFNs production, signalling pathway

## Abstract

Innate immunity is the front-line defense against infectious microorganisms, including viruses and bacteria. Type I interferons are pleiotropic cytokines that perform antiviral, antiproliferative, and immunomodulatory functions in cells. The cGAS–STING pathway, comprising the main DNA sensor cyclic guanosine monophosphate/adenosine monophosphate synthase (cGAS) and stimulator of IFN genes (STING), is a major pathway that mediates immune reactions and is involved in the strong induction of type I IFN production, which can fight against microbial infections. Autophagy is an evolutionarily conserved degradation process that is required to maintain host health and facilitate capture and elimination of invading pathogens by the immune system. Mounting evidence indicates that autophagy plays an important role in cGAS–STING signaling pathway-mediated type I IFN production. This review briefly summarizes the research progress on how autophagy regulates the cGAS–STING pathway, regulating type I IFN production, with a particular focus on the crosstalk between autophagy and cGAS–STING signaling during infection by pathogenic microorganisms.

## Introduction

Innate immunity is the first line of host defense against infectious pathogenic microorganisms. Host cells recognize invading pathogens through interactions between pattern-recognition receptors (PRRs) and pathogen-associated molecular patterns (PAMPs), which can further trigger innate immune responses (Akira et al., 2006). Five different families of PRRs that are speculated to be germline-encoded proteins have been identified. These families include the Toll-like receptors (TLRs), C-type lectin receptors (CLRs), NOD-like receptors (NLRs), retinoic acid-inducible gene (RIG)-I-like receptors (RLRs), and DNA sensors (Kawai and Akira, 2009; Hardison and Brown, 2012; Hoving et al., 2014). Some TLRs and CLRs located on the surface of the cell membrane are the main sensors for extracellular pathogens. The NLRs, RLRs, DNA receptors, and other TLRs are cytoplasmic proteins that help to recognize cytoplasmic PAMPs. The interaction between PRRs and PAMPs can activate multiple signaling pathways in immune cells and induce the production of type I interferons (IFNs) and proinflammatory cytokines to remove pathogenic microorganisms.

Autophagy is an evolutionarily conserved stress response that regulates the decomposition and recycling of superfluous or potentially dangerous cytosolic entities to protect cells from toxic protein accumulation, organelle dysfunction, and pathogen invasion. Given that autophagy has these special functions, a number of studies have attempted to establish a link between autophagy and immune responses. Several relevant proteins in autophagy, such as autophagy-related 16-like 1 (ATG16L1), ATG12-ATG5, BECN1, ULK1, and ATG9, have been confirmed to directly regulate type I IFN antiviral responses. Several key immune regulatory factors involved in type I IFN signaling, such as RIG-I, MAVS, cyclic guanosine monophosphate/adenosine monophosphate synthase (cGAS), and stimulator of IFN genes (STING), are also regulated by autophagy, which in turn can affect type I IFN production. cGAS is an important DNA sensor that detects microbiological DNA, host mitochondrial DNA, or genomic DNA in the cytosol and then triggers a signaling cascade that leads to the production of type I IFNs and inflammatory cytokines. Moreover, Gui et al. recently revealed that autophagy induction is an ancient and highly conserved function of the cGAS–STING pathway that evolutionarily predated the emergence of the type I IFN pathway in vertebrates (Gui et al., 2019). During infection by a pathogenic microorganism, type I IFNs and autophagy can work synergistically or inhibit each other, and they play important roles in regulating pathogen clearance, tissue damage, and immune response. Impaired autophagy contributes to the aberrant activation of STING signaling, thereby leading to uncontrolled inflammation and cell death in sepsis (Hu et al., 2019). It has been demonstrated that STING agonists, such as diABZI, have potent antiviral activity against the respiratory RNA viruses human parainfluenza type 3 virus, rotavirus, and severe acute respiratory syndrome coronavirus 2 (SARS-CoV-2), the causative agent of COVID-19, dependent on the IFN pathway (Zhu et al., 2020; Zhu et al., 2021). Therapeutics such as chloroquine and its derivatives, which inhibit the autophagic pathway, have been suggested for treatment of COVID-19 (Wang et al., 2020), suggesting these is a close connection between autophagy and cGAS–STING signaling in the fight against pathogens. However, other researchers reported that chloroquine has no significant effect on COVID-19. Whether chloroquine helps treat COVID-19 remains controversial, and many of these studies have significant limitations (Gavriatopoulou et al., 2021). The differences in these results may be due to the fact that autophagy is a double-edged sword for the host, especially during infection by some pathogenic microorganisms (Choi et al., 2018). When autophagy is inhibited, pathogenic microorganisms, damaged organelles, and denatured proteins cannot be effectively degraded. However, excessive autophagy causes host immune cell death and leads to disease (Lavandero et al., 2015). In addition, the mechanisms by which different pathogenic microorganisms, and even the same pathogen under different infection conditions, induce autophagy are different (Huang and Brumell, 2014; Choi et al., 2018). Clearly, autophagy is a complex process, which partly explains the differences between studies. In this minireview, to further improve our understanding of the regulatory mechanisms of autophagy and the cGAS–STING signaling pathway, we discuss 1) the multifaceted crosstalk between autophagy and the cGAS–STING signaling pathway in type I IFN production and 2) models of their mutual regulation during pathogen infection, providing a new perspective for studying the mechanisms of host resistance to pathogenic microbial infection.

## Role of Type I Interferons in Infection by Pathogenic Microorganisms

IFNs and their receptors are type 2 alpha-helical cytokines, which are ubiquitous in vertebrates. According to their cellular origin, genetic structure, biological function, and receptor signaling pathways, IFNs can be divided into three different families: type I IFNs, type II IFNs, and type III IFNs. Almost all cells can produce type I IFNs after their PRRs recognize PAMPs. DNA sensors detect microbial DNA, host mitochondrial DNA, or genomic DNA in the cytosol and then trigger a signaling cascade that leads to the production of type I IFNs and inflammatory cytokines. It has been confirmed that the DNA sensors TLR9, cGAS, STING, DHX9, DHX36, DDX41, IFI16, and RNA polymerase III mediate type I IFN production through different mechanisms (Parvatiyar et al., 2012; Zhang et al., 2013). Generally, once PAMPs are recognized, these PRRs induce the downstream signaling pathway and activate transcription factors (such as IRF3 or IRF7) to enter the nucleus to initiate the type I IFN production. Then, the IFN-α/β receptor (IFNAR) recognizes type I IFN and forms the IFNAR1–IFN-α/β–IFNAR2 ternary complex to phosphorylate TYK2 and JAK1. In addition, STAT1/2 is recruited and phosphorylated to form three STAT complexes, which induce downstream IFN-stimulated gene (ISG) expression (Platanias, 2005).

One of the important functions of type I IFNs is activating antiviral immune response. Under virus infection and IFN production, the expression levels of hundreds of ISGs rapidly change to induce an antiviral state within the cell. Translational inhibition is a common mechanism of ISG-mediated antiviral action against hepatitis C virus (HCV), sindbis virus, chikungunya virus, and venezuelan equine encephalitis virus (Schoggins et al., 2011). For example, ISG54 can cooperate with ISG60 to promote apoptosis through the mitochondrial pathway and further interacts with eukaryotic initiation factor 3 to inhibit virus transcription (Stawowczyk et al., 2011). IFN signaling also induces activation of 2′–5′ oligo-adenylate synthetase, catalysis of ATP polymerization, and activation of endonuclease RNase L, which degrades viral mRNA and thus blocks viral protein synthesis upon infection with viruses including encephalomyocarditis virus, reoviruses, semliki forest virus, HCV, and herpes simplex virus 2 (Silverman, 2007; Drahushchenko et al., 2012). IFN-inducible transmembrane protein 3 inhibits viral invasion by blocking the fusion of the virus and the endosomal membrane during infection with influenza A virus or reoviruses but does not inhibit human papillomavirus, cytomegalovirus, and adenovirus infection (Feeley et al., 2011; Anafu et al., 2013; Warren et al., 2014; Ingle et al., 2018). Furthermore, IFNs sensitize cells to apoptosis, which is an important process in antiviral defense (Stark et al., 1998; Seet et al., 2003). In addition, the IFN system is linked to a variety of effector responses of the innate and adaptive immune systems. One characteristic shared by IFN-regulated effector responses is that their activation ultimately results in the elimination of virus-infected cells (Stetson and Medzhitov, 2006). However, during bacterial infection, the function of type I IFNs is still enigmatic. Depending on the bacterium, IFNs exert seemingly opposite and capricious functions. During *Listeria* infection, type I IFNs promote the dissemination and proliferation of the bacteria through accelerating macrophage and lymphocyte apoptosis (Carrero et al., 2004). Moreover, type I IFNs have also been found to aggravate *Francisella tularensis*, *Salmonella*, *Mycobacterium tuberculosis*, and *Listeria* infection through different routes (Storek et al., 2015; Boxx and Cheng, 2016). *Listeria* infection can induce a strong type I IFN response, which makes the host more susceptible to bacteria. *Listeria* that escape into the cytoplasm can be recognized by cGAS, IFI16, and RIG-I, which promote dissemination and proliferation of the bacteria via induction of DAXX and TRAIL (O’Connell et al., 2004; Hansen et al., 2014). IFNAR1 or IRF3 knockout protects the host from *Listeria* infection (O’Connell et al., 2005). However, type I IFNs protect against *Legionella pneumophila*, *Streptococcus pyogenes*, and *Streptococcus pneumoniae* infection (Damjanovic et al., 2014; Boxx and Cheng, 2016; Naujoks et al., 2016). STING recognizes *Legionella pneumophila* in the cytoplasm and triggers IRF3-dependent IFN production, which can inhibit the proliferation of *Legionella* by inducing the expression of intrinsic cellular ISGs (Plumlee et al., 2009; Lippmann et al., 2011; Naujoks et al., 2016). Based on the above, type I IFNs play a key role in controlling the pathogen infection process and maintaining the homeostasis of the organism during interactions between the pathogen and the host. Meanwhile, autophagy, inflammation, and other responses are also involved in pathogen infection.

## Role of Autophagy in Infection Caused by Pathogenic Microorganisms

Autophagy is the process of the degradation of cellular components using autolysosomes. This process is unique to eukaryotic cells. Autophagy is also a highly conserved intracellular degradation process for the elimination of damaged organelles, protein aggregates, and invading pathogens (Levine et al., 2011). According to the differences in the mode of cargo delivery to the lysosome, autophagy mainly includes the following three forms: molecular chaperone-mediated autophagy, microautophagy, and macroautophagy. Macroautophagy is generally referred to as autophagy, which includes the following key steps: vesicle nucleation (formation of the isolation membrane/phagophore), membrane elongation (LC3 lipidation), autophagosome formation, autophagosome fusion with the lysosome to form an autolysosome, and autophagic degradation.

In recent years, research on the interactions between host and pathogen has revealed that autophagy can eliminate invading pathogenic microorganisms and regulate infection-induced innate and adaptive immune responses (Mao and Klionsky, 2017; Jang et al., 2019). The recognition of pathogens is the key step in initiating pathogen elimination. The host PRRs can recognize PAMPs and trigger innate immune responses and autophagy. TLRs recognize pathogenic PAMPs, recruit TRIF or MyD88, and then activate TRAF6, NF-κB, MyD88, and MAPK signaling, which further triggers Beclin-1 to dissociate from the BCL-2 complex and induce autophagy (Choi et al., 2018). In addition, *M. tuberculosis* is recognized by TLR4 and induces (i) the production of various inflammatory cytokines and (ii) SIRT3-dependent autophagy (Kim et al., 2019). Inflammatory cytokines have been found to regulate autophagy. IL-1 promotes autophagy via increasing autophagic flux. TNF-α induces autophagy through the ERK1/2 pathway. Furthermore, these inflammatory cytokines also induce the production of reactive oxygen species and reactive nitrogen species (RNS) and activation of NF-κB and MAPK8/c-Jun kinase, which are involved in the initiation of autophagy (Qin et al., 2011; Yuan et al., 2018). During RNA virus infection, RIG-I and MDA5 take part in IRF3 activation and further induce the production of IFNs, which promotes activation of the VPS34 complex and eIF2α and further triggers autophagy (Talloczy et al., 2002). During DNA virus infection, cGAS competes with Rubicon for Beclin-1 binding, thereby further leading to autophagy and triggering viral DNA degradation to eliminate persistent stimulation (Liang et al., 2014b). However, pathogens have evolved various mechanisms to escape autophagic degradation or to use autophagy to promote their intracellular survival. Some pathogens can inhibit or escape autophagy (Sorbara et al., 2018; Xu et al., 2019). The *Eis* gene of *M. tuberculosis* acts as an N-acetyltransferase and can induce JNK inactivation via acetylating JNK-specific phosphatase and MAPK7. Blocking JNK signaling triggers autophagy inhibition (Shin et al., 2010; Kim et al., 2012). *Burkholderia pseudomallei* uses its type 3 secretion system to escape from the autolysosome into the cytoplasm, where it spreads and replicates (Gong et al., 2011). In contrast, pathogens such as Zika virus (ZIKV), HCV, SARS-CoV, and foot-and-mouth disease virus use autophagy to promote their own survival and infection (Berryman et al., 2012; Wang et al., 2015; Cortese et al., 2017; Miller et al., 2020). HCV induces the formation of autophagosomes but inhibits lysosomal fusion. HCV NS4B helps the virus to replicate in autophagosomes by inducing the expression of Rubicon, which inhibits the maturation of autophagosomes (Guevin et al., 2010). In addition, autophagy is also involved in regulating pathogen-induced inflammation and antigen presentation (Levine et al., 2011; Germic et al., 2019). Above all, autophagy is closely linked to infection by pathogenic microorganisms.

## cGAS–STING Signaling Induces Type I Interferon Production and Triggers Autophagy

**FIGURE 1:**
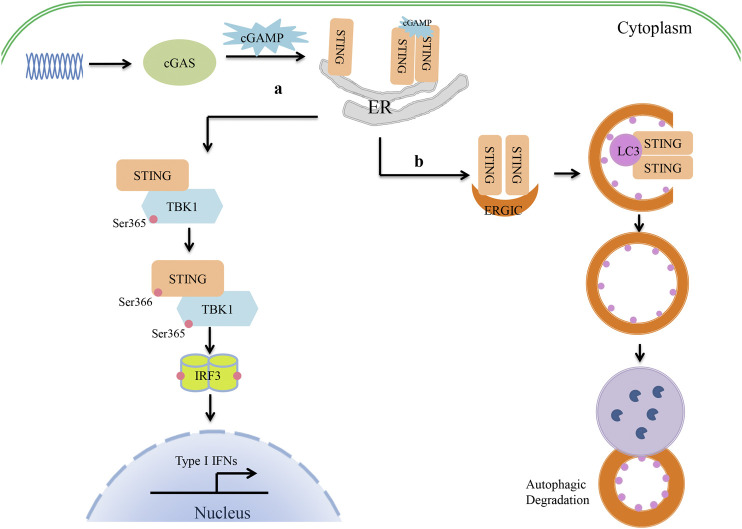
Crosstalk between cGAS–STING signaling and autophagy in type I IFN production. (1) The cyclic guanosine monophosphate/adenosine monophosphate synthase (cGAS)–stimulator of interferon genes (STING) pathway of type I interferon (IFN) production. cGAS recognizes cytosolic DNA and catalyzes the formation of cGAMP. cGAMP serves as a second messenger that binds to the CBD domain of STING. Upon binding with cGAMP, the conformation of STING changes, and oligomerized STING then migrates away from the endoplasmic reticulum (ER). The oligomerization of STING activates TBK1 by phosphorylation at serine 365. The activated TBK1 then phosphorylates the CTT pLxIS motif (Ser366) of STING to recruit IRF3. TBK1 in turn phosphorylates IRF3 and induces the IRF3 dimer to enter the nucleus, where it promotes type I IFN production. (2) cGAS–STING triggers autophagy. Once STING is activated by cGAMP, STING migrates from the ER to the Golgi apparatus via the ER–Golgi intermediate compartment (ERGIC). At the ERGIC, STING has been implicated in autophagy induction. STING-containing ERGIC serves as a membrane source of LC3 lipidation and triggers the formation of autophagosomes. Finally, autophagosomes fuse with lysosomes, where their content is degraded.

Canonical cGAS–STING signaling is an important type I IFN production pathway of innate immunity (Figure 1). cGAS is a broad-spectrum DNA receptor located in the cytoplasm, the inner leaflet of the plasma membrane, and the nucleus (Barnett et al., 2019; Volkman et al., 2019; Hopfner and Hornung, 2020). It can efficiently recognize a variety of DNAs in the cytoplasm, including viral, retroviral, and bacterial DNA and the host’s own DNA (mitochondrial DNA or genomic DNA from dead or damaged cells), and then triggers a signaling cascade that leads to the production of type I IFNs and inflammatory cytokines. cGAS consists of a two-lobed catalytic domain and an extended N-terminal domain. In the absence of DNA stimulation, cGAS is in a self-inhibition state. After binding with single-stranded DNA or double-stranded DNA (dsDNA), the conformation of cGAS changes and it forms a tetramer composed of two cGAS and two DNA molecules. This tetramer catalyzes the reaction of adenosine triphosphate (ATP) and guanosine triphosphate (GTP) to form GMP-AMP (cGAMP) (Gao et al., 2013; Sun et al., 2013). cGAMP serves as a second messenger and leads to STING conformational changes and self-activation by binding to the c-diGMP-binding domain (CBD) of STING (Ablasser et al., 2013; Diner et al., 2013). Cyclic dinucleotides (CDNs) (e.g., c-dGMP, c-dAMP, and cGAMP) produced by bacteria can also directly bind to and activate STING independently of cGAS to induce immune responses (Jenal et al., 2017; Cohen et al., 2019). The activated STING migrates from the endoplasmic reticulum (ER) to the nucleus via the Golgi apparatus and gathers around the nucleus. Upon binding with cGAMP or CDNs, several STING molecules are oligomerized by lateral connections that make the CTT domain of STING better accessible to TANK-binding kinase 1 (TBK1) and phosphorylate TBK1 at serine 365 (Ergun et al., 2019; Shang et al., 2019; Zhang et al., 2019; Zhao et al., 2019). It has been demonstrated that the oligomerization of STING is crucial for the activation of TBK1 and causes it to migrate away from the ER (Shang et al., 2019). The activated TBK1 phosphorylates the CTT pLxIS motif (Ser366) of the STING dimer (Liu et al., 2015). In addition, IRF3 is also recruited to the pLxIS motif of the phosphorylated STING (Shang et al., 2019). Subsequently, the activated TBK1 phosphorylates IRF3 and induces the dimerization of IRF3, which enters the nucleus to promote type I IFN production (Figure 1) (Tanaka and Chen, 2012; Liu et al., 2015). Moreover, cGAS–STING signaling also activates the kinase IKK and stimulates the expression of proinflammatory cytokines through NF-κB. Furthermore, cGAS and STING play important roles in the resistance and elimination of invasive pathogens. It has been suggested that cGAS- or STING-deficient mice fail to produce IFNs and are more susceptible to DNA viruses than wild-type mice, such as HSV-1, murine gamma-herpesvirus virus 68, or vaccinia virus (Li et al., 2013; Ma and Damania, 2016; Liu et al., 2021). Even during RNA virus infection, cGAS- and STING-deficient mice are also more susceptible to the virus than wild-type ones, although the level of IFNs is the same (Schoggins et al., 2014). In addition, the cGAS–STING pathway can be induced by various intracellular bacteria, such as *Listeria*, *Shigella*, *Mycobacteria*, *Legionella*, *Francisella*, *Chlamydia*, *Neisseria*, and group B *Streptococcus*, and participates in the host innate immune response.

Recent studies have found that cGAS–STING is also involved in the regulation of autophagy. Waston et al. found that extracellular *M. tuberculosis* DNA could induce autophagy by activating STING, which suggested there might be an unexpected link between STING and autophagy (Watson et al., 2012). In 2017, Bhatelia et al. reported that STING regulates autophagy flux and mitochondrial turnover through mitophagy (Bhatelia et al., 2017). Moretti et al. (2017) provided evidence that STING is present on autophagosomal membranes and senses live Gram-positive bacteria to mediate ER-phagy and the type I IFN response. Autophagy is induced by STING as follows (Figure 1). Upon binding to cGAMP, the conformation of STING changes. The oligomerized STING then migrates from the ER to the Golgi apparatus via the ER–Golgi intermediate compartment (ERGIC). At the ERGIC, STING is closely associated with the initiation of autophagy induction. The process of STING translocation is dependent on the COP-II complex and ARF GTPases. STING-containing ERGIC serves as a membrane source of LC3 lipidation and subsequently induces autophagosome formation. At last, the autophagosome fuses with the lysosome and degrades the content (Gui et al., 2019). Gui et al. revealed that LC-3-interacting regions (LIRs) of STING could directly interact with LC3 and induce ATG5-dependent non-canonical autophagy. Another very important piece of evidence is that STING from invertebrates induces autophagy, but not IFN, in response to cGAMP stimulation, which implies that the induction of autophagy may be a basic function of the cGAS–STING signaling pathways (Gui et al., 2019). It has therefore been suggested that STING might be an autophagy receptor, but its substrate is still unknown. Clarifying the relationship between STING and p62 will help researchers to understand this process. First, Saitoh et al. found that STING co-localized with p62 and LC3 upon activation (Saitoh et al., 2009). During *M. tuberculosis* infection, STING-induced selective autophagy is dependent on p62 to eliminate *M. tuberculosis* (Watson et al., 2015). However, STING induces non-canonical autophagy, which is dependent on ATG5 but not on p62 (Liu et al., 2019). Further study has shown that STING can be degraded by autophagy in a process that requires TBK1 and p62, which prevents immune damage that would be caused by the continued activation of STING (Prabakaran et al., 2018). Another study has shown that activated STING-induced LC3 lipidation is dependent on WIPI2 and ATG5, but not on ULK and VPS34–Beclin kinase complexes—the most important autophagic signaling complexes (Gui et al., 2019). Moreover, blocking the activity of TBK1, IRF3, and IKK did not affect STING-induced autophagy (Gui et al., 2019). In other cases, bacterial CDNs directly activated STING, leading to ER stress, and then triggered selective autophagy of the ER via the mTOR-BECN1 pathway (Moretti et al., 2017). Therefore, the specific mechanism by which STING regulates autophagy under different conditions should be evaluated carefully. *Drosophila* STING, which lacks the CTT, triggers an innate immune response to restrict microbial pathogens (Martin et al., 2018). It can be inferred that STING is an evolutionarily conserved antimicrobial effector both in arthropods and mammals. Sea anemones, as ancient creatures, can promote autophagy responses through a STING homolog that lacks the C-terminal TBK1 activation domain (Gui et al., 2019). Therefore, triggering autophagy is a primordial function of the cGAS–STING pathway that might have evolutionarily predated the emergence of the type I IFN pathway in vertebrates. STING-dependent autophagy plays an active role in resistance to pathogen infection as IFNs. Liu et al. (2018) reported that ZIKV-induced NF-κB activation promoted the expression of *Drosophila* STING and triggered autophagy to clear the virus. It is noteworthy that during HSV-1 infection, autophagy activation rather than type I IFN signaling seems to be the main effector function of STING in regulating viral infection (Tan and Xia, 2020; Yamashiro et al., 2020). A similar function of STING-dependent autophagy was also found in infections with pathogenic bacteria such as *M. tuberculosis*, *Listeria innocua*, and *Staphylococcus aureus* (Watson et al., 2012; Moretti et al., 2017). It has been shown that STING-induced autophagy helps to clear pathogens from the cytoplasm. However, a recent study has suggested that autophagy induction by STING alone is insufficient to protect mice from HSV-1 infection *in vivo* (Yum et al., 2021). This appears to be inconsistent with most findings given that autophagy often helps to degrade pathogenic microorganisms. STING-S365A mutant mice, which disrupt IRF3 binding and IFN induction but not NF-κB activation or autophagy induction, are resistant to HSV-1 infection. STING-L373A/∆CTT mutant mice, which disrupt IRF3 and NF-κB activation but not autophagy induction, are susceptible to HSV-1 infection. Hence, TBK1 recruitment to STING and subsequent NF-κB activation may play an important role in restricting viral infections. NF-κB-driven expression of CXCL1/2 can result in the recruitment of monocytes and neutrophils to the infected site to perform their antiviral immune function (Iijima et al., 2011). However, owing to the lack of an autophagy-mutant mouse model in this study, the positive role of autophagy in the antiviral response cannot be excluded. The findings also suggest that we may need to pay more attention to the synergistic effects of autophagy and inflammation in future anti-infection studies. Taken together, STING is a key link between type I IFN production and autophagy during infection with pathogenic microorganisms.

## Autophagy Regulates Signal Transduction of cGAS–STING

Constant DNA stimulation results in the aberrant activation of the cGAS–STING pathway and the production of type I IFNs and other cytokines, which further induce immune damage to the host. Generally, moderate autophagy helps to prevent the body from producing excessive inflammatory cytokines. In addition, autophagy plays an important role in regulating the activation of STING to maintain immune homeostasis. Accumulating evidence suggests that several ATG proteins are involved in the cGAS–STING signaling pathway (Table 1). Liang et al. (2014a) found that the autophagy regulatory protein BECN1 directly interacts with cGAS and enhances the autophagy-mediated degradation of cytosolic microbial DNAs to suppress type I IFN production. The ATG16 complex mediates the conjugation of ATG8 phosphatidylethanolamine to the ubiquitin-like molecule LC3 in a process that is essential for the proper formation and function of the autophagosome (Lystad et al., 2019; Fracchiolla et al., 2020). In addition, ATG16 also participates in the regulation of IL-22. It has been found that epithelial IL-22 stimulation leads to the release of cytosolic dsDNA and the consecutive self-activation of the cGAS–STING–IFN-I pathway and necroptosis in the intestinal epithelium, which could be aggravated by autophagy and ER stress deficiency. In small intestinal organoids of villin, the loss of ATG16L1 promotes IL-22-induced IFN-I expression via the STING-dependent recognition of cytosolic dsDNA (Table 1) (Aden et al., 2018). Interestingly, the WD40 domain of ATG16L1 mediates the activation of the STING-dependent V-ATPase-dependent LC3B lipidation onto single-membrane perinuclear vesicles (Table 1) (Fischer et al., 2020). ATG1, also known as serine/threonine UNC-51-like kinase (ULK1/ATG1), is involved in cGAS–STING negative-feedback regulation. cGAS-generated cGAMP can regulate ULK1 activity by separating it from AMP-activated protein kinase. Then, ULK1 phosphorylates STING and suppresses IRF3 function to inhibit the persistent transcription of innate immune-related genes and to prevent inflammatory cytokine dysregulation (Table 1) (Konno et al., 2013). The cargo receptor serves as a bridge to mediate selective autophagy and the cGAS–STING pathway. The K48-linked ubiquitination of cGAS is a recognition signal for p62-dependent selective autophagic degradation, which further inhibits cGAS–STING signaling (Chen et al., 2016). STING degradation following the activation of the cGAS–STING pathway is also mediated by the selective autophagy receptor p62 (p62/SQSTM1). The TBK1-mediated phosphorylation of p62 leads to ubiquitination of STING to trigger autophagy-mediated degradation and the attenuation of type I IFN expression (Table 1) (Prabakaran et al., 2018). In addition, the downstream signaling of cGAS–STING can be regulated by autophagy to affect innate immune signaling. It has been found that ATG9a negatively regulates the cGAS–STING pathway by reducing the assembly of STING and TBK1 following dsDNA stimulation (Saitoh et al., 2009). The activation of IRF3 is the key step to induce type I IFN production, which is precisely regulated by the host immune system. It has been found that the autophagy cargo receptor CALCOCO2/NDP52 promotes IRF3 degradation in a virus load-dependent manner (Table 1). In contrast, the deubiquitinase PSMD14/POH1 prevents autophagic degradation of IRF3 by cleaving the K27-linked poly-ubiquitin chains on IRF3 to maintain IRF3-mediated type I IFN activation (Wu et al., 2021). Thus, autophagy acts as a key factor to provide feedback for the regulation of cGAS–STING-mediated type I IFN production to avoid deleterious consequences.

**TABLE 1:** Regulation of transcription of genes involved in cGAS–STING signaling by autophagy proteins.

Protein	Site	Type of regulation	Function	References
BECN1	cGAS	Directly interacts with cGAS and promotes cytosolic microbial DNA degradation by autophagy	Suppresses type I IFN production	Liang et al. (2014a)
ATG16	Cytosolic dsDNA release	Inhibits IL-22 to suppress cytosolic dsDNA release and cGAS–STING activation; activates the STING-dependent V-ATPase-dependent LC3B lipidation	Suppresses type I IFN production; promotes autophagy	Aden et al. (2018); Fischer et al. (2020)
	STING			
ULK1/ATG1	STING	Phosphorylates STING and suppresses IRF3 function	Suppresses type I IFN production	Konno et al. (2013)
p62	cGAS; STING	Recognizes the K48-linked ubiquitination of cGAS and promotes its degradation by selective autophagy; phosphorylated p62 recognizes STING and promotes its degradation	Suppresses type I IFN production	Chen et al. (2016); Prabakaran et al. (2018)
ATG9a	STING	Reduces the assembly of STING and TBK1	Suppresses type I IFN production	Saitoh et al. (2009)
CALCOCO2/NDP52	IRF3	Promotes IRF3 degradation in a virus load-dependent manner	Suppresses type I IFN production	Wu et al. (2021)

## Conclusion

As an important sensor of innate immunity, cGAS–STING controls infection caused by pathogenic microorganisms in multiple ways, including the induction of type I IFN production, the activation of inflammatory responses, and triggering autophagy. In this review, we discussed the relationships between the cGAS–STING pathway and autophagy in type I IFN production. It is indisputable that the activation of STING is crucial to type I IFN production and autophagy activation. In turn, autophagy strictly regulates the activation of cGAS–STING in a variety of ways, such as promoting the degradation of cGAS or STING, binding to adaptor proteins, and regulating the post-transcriptional modification of key molecules. Several ATGs and autophagy cargo receptors, such as ATG1, ATG9a, ATG16L1, p62, and CALCOCO2/NDP52, have been found to participate in this process (Figure 1). With more in-depth research, it has become obvious that the autophagy machinery is not as simple as previously thought. Autophagy-monitoring and autophagy-deficient mouse models have yielded a huge amount of data about the functions of autophagy in mammals (Sandoval et al., 2008; Cheong et al., 2011; Chew et al., 2015; Tsuboyama et al., 2016). The combination of these STING- or cGAS-deficient mouse models will likely help researchers discover other molecules and new mechanisms involved in the regulation of autophagy. The study of cGAS–STING and autophagy crosstalk will deepen our understanding of the regulation of IFNs and the interactions between pathogens and hosts. This review provides a new perspective for studying host resistance mechanisms to pathogenic microbial infection. Since cGAS–STING plays a critical role in both IFN production and autophagy, it may serve as a therapeutic target to stimulate host resistance to pathogens.
